# Red cell distribution width and preeclampsia: a systematic review and meta-analysis

**DOI:** 10.1186/s40885-019-0119-7

**Published:** 2019-07-15

**Authors:** Ishag Adam, Theonest K. Mutabingwa, Elfatih M. Malik

**Affiliations:** 1 Faculty of Medicine, University of Khartoum, Khartoum, Sudan; 2 Hubert Kairuki Memorial University, Dar es Salaam, Tanzania

**Keywords:** Preeclampsia, Pregnancy hypertension, Red cell distribution width, Meta-analysis, Sudan

## Abstract

**Background:**

Preeclampsia is a serious pregnancy-related disease which may lead to adverse health effects to the mother and fetus. Besides many publications on the association of red cell distribution width (RDW) and preeclampsia, there has been no published meta-analysis. This necessitated the present systemic review and met-analysis to assess the RDW in relation to preeclampsia.

**Methods:**

Preferred Reporting Items for Systematic Reviews and Meta-Analyses guideline was followed. Relevant published studies were searched in PubMed, Cochrane library, Google scholar, Scopus, Embase and CINAHL using the term “Preeclampsia OR eclampsia AND red cell distribution width OR red blood cells). Modified Newcastle – Ottawa quality assessment scale was used for critical appraisal of retrieved studies. Pooled Meta logistic regression was computed using OpenMeta Analyst software. Subgroup and meta-regression methods were performed to analyse the heterogeneity.

**Results:**

Eleven case control studies were included in the met-analyses with a total of 951 cases (preeclampsia) and 2024 controls. The mean (SD) of the RDW level was significantly higher in women with preeclampsia compared to controls [15.10 (2.48) % vs. 14.26(1.71) %, *P* < 0.001]. The mean difference was 0.85, 95% CI = 0.26–1.43. Due to a high heterogeneity (I^2^ = 90.45, *P* < 0.001), the continuous random effect model was used.

Eight studies compared RDW level in the mild (*N* = 360) with severe cases (*N* = 354) of preeclampsia. The RDW level was significantly higher in women with severe preeclampsia compared to those with mild preeclampsia [15.37 (2.48) % vs. 14.037(1.79) %, *P* < 0.001]. The mean difference was 1.07, 95% CI = 0.45–1.70. Since there is a high heterogeneity [I^2^ = 76.67, *P* < 0.001], the continuous random effect model was used.

Through the met-regression model, except for the region of the study (*P* < 0.001), none of investigated variables (age, parity, quality of the study) was significantly associated with the investigated heterogeneity. The outliers (3studies) were removed to reduce the heterogeneity. The pooled meta-analysis of the remaining 8 studies showed a significant difference in the RDW between preeclamptic women compared with the controls. The mean difference was 0.93, 95% CI = 0.56–1.31, *P* < 0.001. Because of heterogeneity [I^2^ = 69.6, *P* = 0.002], the continuous random effect model was used.

**Conclusion:**

RDW level was significantly higher in women with preeclampsia compared to controls. Similarly, women with severe preeclampsia had significantly higher RDW than those with the mild form.

**Electronic supplementary material:**

The online version of this article (10.1186/s40885-019-0119-7) contains supplementary material, which is available to authorized users.

## Background

Preeclampsia is the occurrence of hypertension and proteinuria in the second half of pregnancy (i.e. after the 20 weeks of gestation) in women who had no previous hypertension or proteinuria. With the prevalence of around 3–8% [1, 2], it is a serious pregnancy-related complication that almost always leads to adverse effects to both the mother and fetus [1, 3]. Whereas most cases of preeclampsia are mild and symptomless, it may also occur in severe form, presenting as HELLP-syndrome (hemolysis, elevated liver enzymes, low platelets), cerebral manifestations and eclampsia [4]. Although the exact pathophysiology and pathogenesis of preeclampsia is not completely understood, it is theorized that poor/abnormal placentation in early pregnancy leads to placental ischemia and release of vasoactive substances with consequent endothelial activation and dysfunction [5].

Red cell distribution width (RDW) is one of the suggested helpful markers of systemic inflammatory response [6] besides changes in the levels of hematological parameters e.g. neutrophil-lymphocyte ratio, platelet-lymphocyte ratio, mean platelet volume (MPV), and platelet count (PCT). Furthermore, recent studies have shown these potential markers to be of prognostic as well as clinical predictive values in various benign and malignant diseases including coronary artery disease, inflammatory diseases, preeclampsia and gynaecological or gastrointestinal malignancies [6–10]. RDW which is a numerical measure of the cell size variation of circulating erythrocytes has recently been reported as a strong and independent predictor of adverse outcomes in the diffident diseases and cancers [11–13].

Despite many published articles on the association of RDW and preeclampsia [14–25] there has been no published meta-analysis on this aspect to the best of our knowledge. The present systemic review and met-analysis on RDW in relation to preeclampsia was therefore conducted to bridge knowledge gap. Its findings will possibly represent high grade scientific evidence base that may be used for policy formulation and/or for updating clinical case management.

## Materials and methods

### Searching strategies

**Fig. 1:**
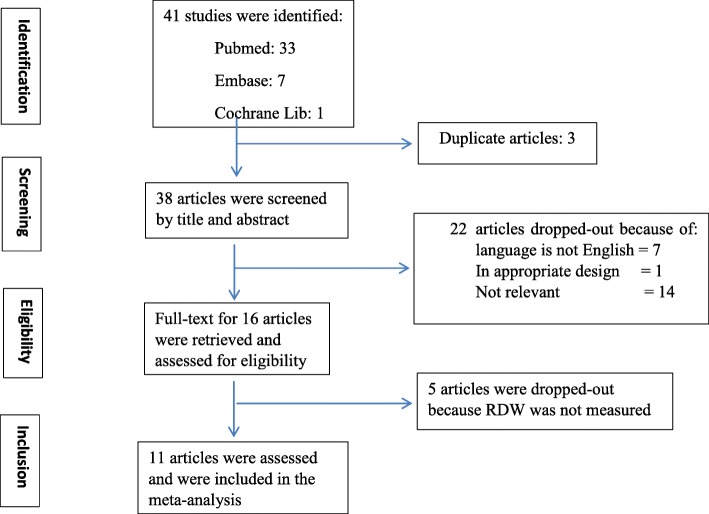
Flow diagram through study search and inclusion

Preferred Reporting Items for Systematic Reviews and Meta-Analyses (PRISMA) guideline was followed in undertaking this systematic review and meta-analysis [26]. Briefly, literature search was performed in PubMed, Cochrane library, Google scholar, Scopus, Embase and CINAHL. In PubMed the search terms used were: Preeclampsia [MeSH] OR eclampsia [MeSH] OR hypertension in pregnancy AND red cell distribution width [MeSH] OR red blood cells [MeSH]. All studies published up to August 15/2018 were retrieved and assessed for eligibility based on the inclusion/exclusion criteria. Titles and abstracts of identified and retrieved papers were exported to Endnote whereby duplicates were removed. Full texts of retrieved articles were assessed and corresponding reference lists checked to identify further relevant articles (Fig. 1).

### Inclusion criteria

The inclusion criteria were: original papers published in English addressing human pregnancy, preeclampsia investigated on maternal side using strict definition and RDW investigated and reported.

### Exclusion criteria

Topic reviews, case report of less than five cases, in vitro or animal studies, posters or conference abstracts, only pregnancy-induced hypertension studied and studies without healthy pregnant women as controls.

### Quality assessment and data collection

Included studies were assessed using Joanna Briggs Institute Meta-Analysis of Statistics Assessment and Review Instrument (JBI-MAStARI) [27]. Modified Newcastle Ottawa quality assessment scale for cross sectional and case-controls studies was used to assess quality [28], which has a total score of nine (9). A study was considered high quality if it scored 7 and above, and medium if the score was 5 out of 9 (Table 1).

**Table 1:** Ottawa rating for included studies: (* OR ** means criteria fulfilled/Maximum score =9)

Study	Is the case definition adequate?	Selection	Selection of Controls	Definition of Controls	Comparability	Ascertainment of exposure	Outcome	Non-response rate	Total Score
		Representativeness of the cases			Comparability of cases and controls		Same method of ascertainment		
Çintesun, et al	*	*	*	*	**	*	*	–	8
Elgari, et al	*	*	*	*	**	*	*	–	8
Prasmusinto, et al	*	*	*	*	*	*	*	–	7
Yücel and Ustun	*	*	*	*	**	*	*	–	8
Reddy, et al	*	*	*	*	**	*	*	–	8
Sen-Yu W, Chao X.	*	*	*	*	**	*	*	–	8
Yılmaz, et al	*	*	*	*	**	*	*	–	8
Avcıoğlu, et al	*	*	*	*	*	*	*	–	7
Abdullahi, et al	*	*	*	*	**	*	*	–	8
Kurt, et al	*	*	*	*	**	*	*	–	8
Huang, et al	*	*	*	*	**	*	*	–	8

Two reviewers (IA and EMM) independently assessed the quality of each article for inclusion in the review. Any disagreement between the reviewers was resolved through discussion with the third independent reviewer (TM).

#### Data extraction

The most important relevant information that was extracted was transcribed into a table requiring the authors’ name, year of publication, study location, number of cases and controls, level of RDW in both the cases and controls, number of mild and severe preeclampsia. For additional detailed information see Additional files 1 and 2. Whenever the median (range) or median (inter-quartile) were reported these were transformed into mean (SD) as previously described [29, 30].

## Statistical methods

### Data analysis and heterogeneity assessment

Open Meta Analyst software for Windows [31, 32] was used to perform all meta-analyses of the difference in the level of RDW between cases and controls. The heterogeneity of included studies was evaluated using Cochrane Q and the I^2^. Cochrane Q with *P* < 0.10 and I^2^ > 50 was taken as standard to indicate the presence of heterogeneity of included studies [33]. Based on the results, the random effects or fixed model was used to combine included studies. A sub-group analysis was also done to investigate the difference between RDW level in mild and severe forms of preeclampsia.

Subgroup and meta-regression methods were used to investigate potential sources of heterogeneity using the following variables: the difference in maternal age, parity, study quality and the study geographic region (Turkey vs. outside Turkey).

### Ethical considerations

PRISMA guideline recommendations were used and strictly abided with during this systematic review and meta-analysis. Being a systematic review and meta-analysis, ethical approval was not required.

## Results

The search strategy identified 41 articles that reduced to 38 articles after applying inclusion and exclusion criteria. Twenty two more studies were excluded based on title and/or abstract, and another 5 excluded after retrieving the full-text of articles (Fig. 1).

Eleven case-control studies fulfilling the inclusion criteria and were used in the met-analysis [14, 15, 17–19, 21–24, 34, 35]. All the 11 studies were of high quality with an Ottawa rating of 7 and 8 (Table 1). Of the eligible studies, 5 (45%) [15, 17, 18, 21, 23] and 2(18%) [22, 24] studies were conducted in Turkey and Sudan respectively. One (9%) study each was conducted in China, India, Taiwan and Indonesia.

**Fig. 2:**
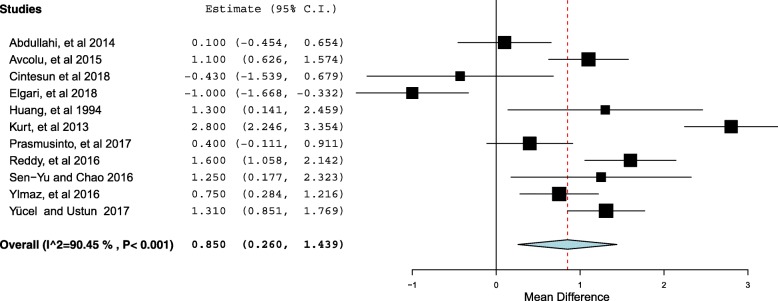
Forest plot comparing the RDW in women with preeclampsia and controls

There were a total of 951 cases (preeclampsia) and 2024 controls. The number of the cases per study ranged from 21 [35] to 143 [14], while the controls ranged from 50 [23] and 911 [14]. The median and variance was reported in three studies [18, 19, 21] and this was transformed to the mean (SD) using the prescribed formula [29, 30]. The mean (SD) of the RDW level was significantly higher in women with preeclampsia compared to controls [15.10 (2.48) % vs. 14.26(1.71) %, *P* < 0.001]. The mean difference was 0.85, 95% CI = 0.26–1.43 (Fig. 2). The I^2^ test result showed a high heterogeneity (I^2^ = 90.45, *P* < 0.001). Therefore the continuous random effect model was used.

**Fig. 3:**
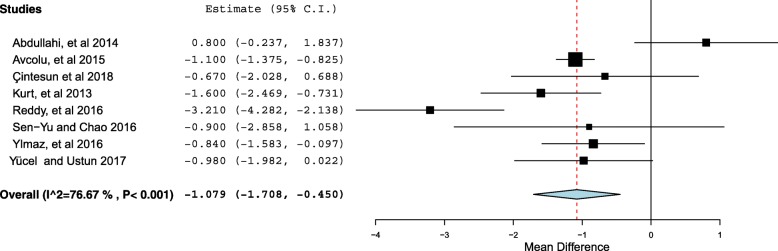
Forest plot comparing the RDW in women with mild and severe preeclampsia

Eight studies compared RDW level in the mild (*N* = 360) with severe cases (*N* = 354) of preeclampsia [14, 15, 17, 18, 21, 23, 24, 34]. There were 360 and 354 women with mild and severe preeclampsia, respectively. The RDW level was significantly higher in women with severe preeclampsia compared to those with mild preeclampsia [15.37 (2.48) % vs. 14.037(1.79) %, *P* < 0.001] (Additional file 2). The mean difference was 1.07, 95% CI = 0.45–1.70 (Fig. 3). Since the I^2^ test result showed high heterogeneity [I^2^ = 76.67, *P* < 0.001], the continuous random effect model was used. Only one study investigated the RDW in early and late preeclampsia and showed the RDW level to be significantly higher in women with early preeclampsia [19].

### Subgroup and meta-regression

**Fig. 4:**
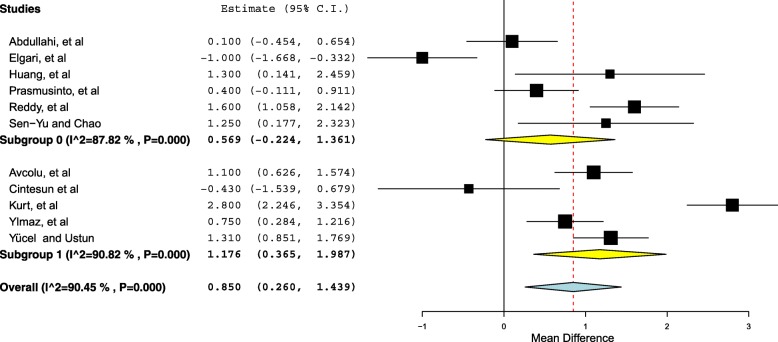
Forest plot of subgroup analysis according to the regions

In view of the observed high level of heterogeneity between studies, results of meta-regression are given in Table 2. Through the regression model, except for the region of the study (*P* < 0.001), none of investigated variables was significantly associated with the investigated heterogeneity. Furthermore, we investigated the RDW and study region. A significant difference was found between studies conducted in Turkey and studies conducted in other regions. The mean difference of the RDW in the studies conducted in Turkey was 1.17, 95% CI = 0.36–1.98, *P* = 0.004 (Fig. 4).

**Table 2:** Results of meta-regression for the RDW and preeclampsia

Covariate	Coefficient	95% confidence interval	Standard error	*P*
Age	– 0.173	– 0.494– 0.147	0. 164	0.290
Parity	– 0.680	– 1.55– 0.195	0.446	0.128
Quality of the studies	−0.632	−1.93–0.67	0.666	0.343

**Fig. 5:**
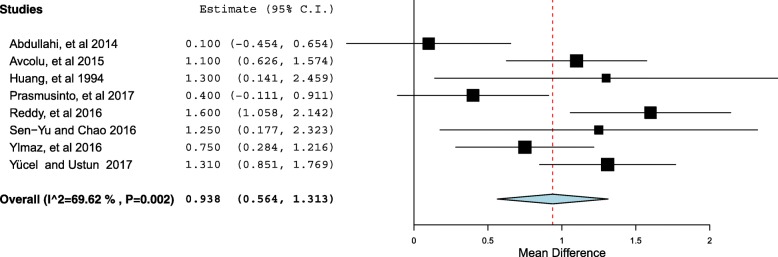
Forest plot of Forest plot comparing the RDW in women with preeclampsia and controls after removing the outliers

Then the outliers (3studies) were removed [14, 17, 23] to reduce the heterogeneity. The pooled meta-analysis of the remaining 8 studies [14, 15, 18, 19, 21, 24, 34, 35] showed a significant difference in the RDW between preeclamptic women compared with the controls. The mean difference was 0.93, 95% CI = 0.56–1.31, *P* < 0.001(Fig. 5). Because of heterogeneity [I^2^ = 69.6, *P* = 0.002], the continuous random effect model was used.

## Discussion

The main finding of the current met-analyses was a high level of RDW in women with preeclampsia compared to controls. With regard to severity, women with severe preeclampsia had significantly higher RDW than those with mild preeclampsia. Also, a recent study reported significant association between RDW and newly diagnosed essential hypertension [36]. As mentioned above RDW is reported as useful indicator in cardiovascular diseases and inflammatory process [11–13].

The RDW is a readily available non-expensive hematologic parameter reflecting a variation in erythrocyte volume (anisocytosis). It is a component of the full blood picture which is a common investigation requested by clinicians attending patients.

Findings of this systematic review corroborates those of previous reports [11–13], thereby strengthening its consideration for use as a marker for preeclampsia/eclampsia in clinical case management.

Although the exact mechanism behind the high level of RDW is not yet fully understood, high-RDW levels might be a reflection of increased inflammation [10] or defective erythropoiesis or hemolysis [23]. Inflammation (inflammatory cytokines) could impair iron metabolism that shorten red blood cells lifespan with increased RDW as consequence [37–39]. Interestingly, other inflammatory markers such as C-reactive protein and erythrocyte sedimentation rate have also been reported to be associated with RDW [40]. Inflammation might inhibit the production or response to erythropoietin, or might directly shortened red blood cell survival [41]. The pro-inflammatory cytokines were positively and inversely associated with erythropoietin concentration in older adults [42].

Besides inflammation, oxidative stress and oxidative damage might also contribute to anisocytosis and elevated RDW [43]. The inflammatory process, oxidative stress (which are features of preeclampsia) and hypoxia with red blood cell destruction may explain the increased level of RDW in preeclampsia especially the severe form of the disease [44, 45].

### Limitations

All of these studies were conducted after the occurrence of the disease itself (preeclampsia) making it to clearly define the cause-effect process. Furthermore the cut off of RDW was not determined. Study sizes of most studies were relatively small and heterogeneous even though we investigated some heterogenic factors. All this calls for longitudinal studies on RWD in early pregnancy with close follow up.

## Conclusion

Based on this review, there was significantly higher level of RDW in women with preeclampsia compared to controls. Women with severe preeclampsia had significantly higher RDW than those with mild forms strengthening consideration of its use as a clinical marker in clinical case management.

## Additional files

Additional file 1: Characteristics of the mild and severe preeclampsia studies included in the systematic review and meta-analysis. (XLSX 13 kb)

Additional file 2: Characteristics of all the studies included in the systematic review and meta-analysis. (XLSX 11 kb)

## Abbreviations

CI: Confidence Interval
IDA: Iron deficiency anemia
OR: Odd ratio
RDW: Red cell distribution width
WHO: World Health Organization
